# Reimagining accessibility in adolescent mental health: PESoMind, a dance-based framework for wellness promotion

**DOI:** 10.3389/fpubh.2026.1847349

**Published:** 2026-06-12

**Authors:** Tara Srinivasan, Afshan Khan

**Affiliations:** 1 Westlake High School, Austin, TX, United States; 2 Austin Family Psychiatry, Austin, TX, United States; 3 Department of Psychiatry & Behavioral Sciences, Dell Medical School, The University of Texas at Austin, Austin, TX, United States

**Keywords:** accessibility, adolescent mental health, dance intervention, digital health, health equity

## Abstract

Adolescent mental health challenges are rising globally, yet structural barriers around cost, geographic limitations, provider shortages, and stigma prevent significant portions of the population from accessing traditional services. This Perspective argues for a paradigm shift toward accessible, multi-modal, low-barrier interventions that complement clinical care. We introduce PESoMind, a dance-based framework intentionally designed for accessibility: requiring no specialized training, minimal time (10–15 min expert-led or under 5 min self-directed), and deliverable via digital platforms. Drawing from a proof-of-concept workshop at SXSW EDU 2025 (*N* = 12 adult stakeholders), we illustrate how integrating physical activity, emotional expression, social connection, and mindfulness through dance creates multiple entry points for diverse adolescents. The digital platform (jivetothrive.com) utilizing this framework now reaches 500+ students, illustrating scalability potential. We discuss implications for public health practice, arguing that equity-focused innovation requires rethinking not just what interventions we offer, but who can access them and under what conditions. This framework offers a model for designing mental health promotion tools that meet adolescents where they are, both literally and figuratively.

## The adolescent mental health access crisis

Adolescent mental health challenges are rising globally, with increasing rates of anxiety, depression, and suicide among young people (1, 2). While clinical interventions remain essential, we face a fundamental access problem: most adolescents who need mental health support do not receive it.

The barriers are well-documented and structural. Cost creates exclusion, with many families unable to afford therapy even with insurance. Geographic limitations leave rural adolescents without local providers. Provider shortages mean months-long waitlists in areas that have services at all. Stigma prevents help-seeking, particularly among marginalized communities where mental health challenges may be culturally misunderstood or dismissed (3).

Traditional responses to this access crisis have focused on expanding clinical capacity: training more therapists, increasing school counselors, expanding telehealth. These efforts are necessary but insufficient. They assume the problem is simply supply and that if we had enough clinicians, all adolescents would access care. This assumption ignores that many adolescents prefer non-clinical approaches, that stigma and distrust prevent help-seeking in some communities (3), and that even with perfect access, clinical models cannot serve everyone who would benefit from mental health promotion.

Public health frameworks increasingly recognize the value of preventive and wellness-oriented interventions that complement clinical care (4). Yet most existing interventions, even those positioned as “accessible”, carry hidden barriers. Group therapy requires transportation and fixed schedules. Mindfulness apps assume smartphone access and digital literacy. School-based programs depend on educator training and institutional buy-in. Each requirement excludes another segment of the adolescent population.

This Perspective argues for a different approach: designing interventions with accessibility as the foundational principle, not an afterthought. We introduce PESoMind as an illustrative case of accessibility-first design, showing how deliberately addressing barriers can create tools that reach adolescents currently excluded from mental health promotion.

## What this perspective offers (and what it doesn't)

This manuscript presents a framework for making mental health support accessible to adolescents who can't reach traditional services. The core contribution is the accessibility-first design logic around how we intentionally built PESoMind to work within the constraints most teens face. The pilot data included here come from a workshop with adult educators and youth advocates, not adolescents themselves. These data serve one specific purpose: showing that the framework is feasible to implement and acceptable to the adults who work with teens daily.

This is not an effectiveness study. This study cannot claim PESoMind improves adolescent mental health because PESoMind has not been scientifically tested with adolescents yet. What this study does claim is that the design addresses real barriers, the components have theoretical and empirical support from existing research, and stakeholders who understand adolescent wellness find it promising enough to warrant further testing. The contribution here is conceptual: reimagining intervention design when accessibility is the starting point, not an afterthought.

## Rethinking intervention design: accessibility as foundation

Many mental health interventions are designed for efficacy first, with accessibility considered later. If that intervention cannot reach populations who need it most, its clinical efficacy is irrelevant to health equity. Accessibility-first design inverts this sequence as it looks to create interventions optimized for the actual conditions under which adolescents access mental health tools. That approach needs to address the following constraints:

**Time constraints**: Interventions must fit within existing schedules such as advisory periods or lunch breaks, requiring 15 min or less rather than hour long sessions.

**Cost barriers**: Interventions cannot require payment, specialized equipment, or transportation.

**Training requirements**: Activities must be deliverable by non-specialists with minimal preparation.

**Space limitations**: Interventions must work in classrooms, homes, or any area where participants can extend their arms.

**Diverse entry points**: Single-modality interventions (e.g., talk-only) exclude those for whom that modality is inaccessible or unappealing.

These constraints are not limitations. Instead, they provide the requirements for equitable interventions.

## PESoMind: an accessibility-first framework

PESoMind emerged from 12 years of classical and contemporary dance training combined with direct observation of access barriers facing adolescents seeking mental health support. As the name indicates, the framework integrates four evidence-based coping strategies: *P*hysical activity, Emotional expression, Social connection, and Mindfulness—delivered through dance movements drawn from diverse cultural traditions (Bharatanatyam, hip-hop, contemporary).

The framework operationalizes accessibility through specific design choices:

Brief sessions: Expert-led activities run 10–15 min, fitting within advisory periods or wellness breaks. Self-directed practice via a digital platform called jivetothrive.com takes under 5 min, enabling daily engagement without schedule disruption.

No specialized training: Facilitators need no dance background. All movements are accessible regardless of prior experience as they are framed as wellness practice rather than performance.

Minimal space requirements: Any area where participants can extend arms suffices which includes classrooms, bedrooms, or community centers. No dedicated studio is needed.

Multiple entry points: The four-component structure (physical-emotional-social-mindfulness) provides diverse pathways for engagement. Dance-interested youth can engage through movement exploration. Those drawn to emotional expression find outlets through guided expressive sequences. Socially oriented adolescents can connect through partner activities. Mindfulness-focused participants can engage through breath work and present-moment awareness. This “something for everyone” approach increases reach across varied adolescent populations.

Cultural responsiveness: Drawing from multiple dance traditions (Indian classical, hip-hop, contemporary) signals inclusivity and allows cultural identification for diverse participants.

Hybrid delivery: Brief experiential workshops build skills and community; digital platform (jivetothrive.com) enables ongoing self-directed practice, removing traditional barriers of cost, transportation, and scheduling.

## Proof-of-concept: SXSW EDU 2025 workshop

To test initial acceptability and feasibility, we delivered a 90-min experiential workshop at SXSW EDU 2025 in Austin, Texas. Approximately 60 educators, youth advocates, and mental health professionals participated. While adult stakeholders rather than adolescents themselves, this population provided valuable perspectives on the intervention's acceptability and feasibility in educational settings.

Workshop activities integrated the following: (1) guided movement sequences emphasizing physical activation (20 min); (2) expressive movement focused on emotional awareness and release (20 min); (3) partner and group exercises promoting social connection (20 min); and (4) mindful movement emphasizing present-moment awareness (20 min). All movements were accessible regardless of prior dance experience.

### Ethical considerations

This workshop was conducted as part of an educational conference session at SXSW EDU 2025. Participation was voluntary, and data collection involved anonymous post-workshop surveys with no personally identifying information. Under institutional policy, this activity was determined to be exempt from Institutional Review Board review as it constituted educational program evaluation in a public conference setting with minimal risk to participants and no collection of identifiable private information.

Twelve participants completed immediate post-workshop assessments using Numerical Rating Scales (0–10) across four dimensions: energy level, emotional state, social connectedness, and mindfulness. Participants also provided qualitative feedback and indicated whether they would recommend the intervention to peers.

### Outcomes

Participants reported consistently high ratings across all measured dimensions: energy (*M* = 9.17, *SD* = 0.83), mindfulness (*M* = 8.75, *SD* = 0.87), social connectedness (*M* = 7.25, *SD* = 1.07), and emotional state (*M* = 8.58, *SD* = 0.90). All participants (100%) indicated they would recommend the intervention to peers.

The qualitative themes aligned with the four PESoMind dimensions.

- Physical energy: Participants described “increased energy,” “feeling more awake,” and appreciated that movements were accessible without prior dance experience.
- Emotional expression: Many highlighted “release,” “emotional freedom,” noting the workshop provided rare opportunity for emotional expression in a professional setting.
- Social connection: Group activities generated themes of “feeling connected,” “sense of community,” with several noting that movement-based connection felt different from verbal interaction.
- Mindfulness: Participants reported “being fully present,” “focus on the now,” contrasting this with typical conference multitasking.

These preliminary findings establish initial acceptability among adult stakeholders and suggest that the framework resonates with those who work directly with adolescents.

### Study limitations

This pilot has significant methodological limitations that need to be stated upfront. The sample size is extremely small (*N* = 12 of approximately 60 workshop participants), and participants were adults, who were educators and youth advocates, not adolescents. The choice of adult stakeholders for this initial pilot was because they work with teens daily and could provide feedback on implementation feasibility, but this creates a fundamental validity problem–this study cannot make claims as to how adolescents would exactly respond to PESoMind based on data from adults.

The study design lacks basic controls: no comparison group, no baseline measurements, no validated assessment instruments. The 0–10 rating scales used were created for this workshop specifically, not drawn from established measures. Participants completed surveys immediately after the workshop, so there is no information about whether acceptability would hold over time or whether anyone would actually use the framework again after leaving the room. The 20% response rate (12 of 60 participants) indicates that the response is from a self-selected group who were motivated enough to complete a survey and probably the people who liked the workshop most.

All of this means the findings are exploratory and illustrative, not generalizable. The data show that these adults found the framework acceptable in this context. That's useful information for deciding whether to invest in more rigorous testing, but it doesn't constitute evidence that PESoMind works for adolescents or improves mental health outcomes.

## Digital platform: reach vs. engagement

This framework has been incorporated in a digital platform called jivetothrive.com. The jivetothrive.com platform addresses the scalability challenges faced by traditional mental health interventions by enabling self-directed access. The platform provides options for brief practice sessions (under 5 min) or extended sessions (10–15 min). Users can access the framework during any available window, such as before school, during breaks, or at home, eliminating traditional barriers of transportation, scheduling, and ongoing facilitator costs.

The platform has recorded over 500 unique user sessions since launch, indicating reach as people are finding and accessing the resource. However, this reach doesn't inform us about engagement or effectiveness of the platform, as the platform doesn't collect detailed behavioral metrics, partly because privacy was prioritized over analytics. While the session logs indicate that over 500 users have engaged in activities, it is unclear whether they tried the movements or found it useful. As such, the platform is still in its infancy to demonstrate true scalability.

True scalability requires understanding the full pathway: can people find it? (Reach) → Do they try it? (Adoption) → Do they keep using it? (Engagement) → Does it help? (Effectiveness). Right now, the data suggests early success with the first step. Future research will focus on acquiring behavioral metrics such as frequency of use, session duration, return visits, and eventually establish whether platform use is associated with any measurable outcomes. Until then, the platform serves as proof-of-concept for digital delivery but cannot make claims about scalable impact.

The dual delivery model which includes brief experiential workshops paired with ongoing digital access, leverages strengths of both approaches. Workshops build skills, community, and initial motivation while the platform supports continued practice without barriers. This combination may address sustainability challenges that plague single-modality interventions, though empirical testing is required to confirm this hypothesis.

## Implications for public health practice

PESoMind illustrates that accessibility constraints can drive innovation rather than limit it. It exemplifies the notion that when we design for underserved constituents such as adolescents in under-resourced schools or rural areas or families unable to afford therapy, we create tools that work for everyone. As such, the framework offers several contributions to public health practice:

- Complement, not compete with clinical care: School counselors and health educators can integrate brief PESoMind sessions into wellness programming, expanding reach without replacing essential clinical services requiring therapeutic support.
- Immediate implementation potential: Minimal barriers (no specialized training, no dedicated space, brief time commitment) enable immediate adoption by schools and community organizations addressing health equity concerns when traditional interventions can take months or years to initiate.
- Multiple implementation models: The framework supports varied delivery approaches-school advisory periods, physical education or after-school programs, allowing local adaptation.

## Critical research questions

This Perspective establishes initial feasibility but leaves fundamental questions unanswered. Future research must address:

- Establish effectiveness with adolescent populations: Randomized controlled trials with adequate adolescent samples (*N* = 100+) comparing PESoMind to alternative interventions are essential.
- Sustain engagement and outcomes: Longitudinal designs must evaluate whether initial acceptability translates to sustained engagement and durable benefits.
- Establish moderators of effectiveness: Examining demographic factors, baseline mental health status, cultural background, and implementation settings will illuminate for whom PESoMind works best, and under what conditions.
- Evaluate across diverse populations: Trials must intentionally recruit underserved populations to evaluate effectiveness across socioeconomic, geographic, and cultural contexts.
- Compare access vs. longer and more intensive programs: Research must evaluate whether brief, accessible interventions achieve comparable outcomes to longer, more intensive programs to inform resource allocation.

## Cultural responsiveness and adaptation

PESoMind integrates movements from Bharatanatyam (classical Indian dance), hip-hop, and contemporary dance, illustrating the framework's capacity for cultural adaptation. The structure is intentionally designed to enable communities to reshape movement sequences using dance traditions meaningful to their specific adolescent populations. This adaptability is central to cultural responsiveness: rather than prescribing universal movements, the framework provides a foundation that communities can customize to their cultural contexts.

The movements currently incorporated reflect the developer's lived experience and training, but adolescents in different cultural contexts will have their own movement traditions that carry meaning for them. The framework structure (integrating physical activity, emotional expression, social connection, and mindfulness through movement) can remain consistent while the specific movements change. A group in rural Appalachia might draw from square dance traditions. Adolescents in West African communities might incorporate traditional dance forms from their heritage. The goal is for communities to use movement languages that already have cultural meaning for their populations, rather than adopting a pre-determined set.

Using movements from multiple dance traditions, especially traditions outside the developer's heritage, risks cultural appropriation: taking cultural practices without understanding their context or significance. While Bharatanatyam represents the developer's cultural heritage and hip-hop and contemporary dance reflect years of formal training, future implementations of PESoMind require participatory design processes where communities decide which movements carry meaning for their adolescents.

Community co-creation might involve working with local dance instructors or cultural leaders to identify meaningful movement forms and consulting adolescents themselves about what kinds of movement feel authentic. The digital platform (jivetothrive.com) currently demonstrates movements from the developer's practice, but broader implementation would benefit from community-contributed variations showing how different groups have adapted the framework to their cultural contexts.

The current framework reflects one person's cultural background and movement training. This is appropriate for a proof-of-concept pilot, but likely insufficient for widespread implementation. Systematic cultural adaptation frameworks have not yet been established, and whether adolescents from different cultural backgrounds find the current movements meaningful or alienating remains untested. This requires attention before claiming the intervention is truly accessible across diverse populations. Cultural accessibility is as important as financial or geographic accessibility.

## Theoretical foundation

PESoMind draws on three established frameworks: Movement-based and expressive therapies, multi-component intervention theory, and preventive health promotion models.

Movement-Based and Expressive Therapies: Movement-based interventions recognize that physical activity and emotional processing are interconnected (5). Dance allows intentional movement to access emotions that may be difficult to verbalize. For adolescents who struggle with talk-based therapy or lack vocabulary to describe internal experiences, movement provides an alternative pathway for emotional expression and regulation.

Multi-Component Integration: Most adolescent mental health interventions target single mechanisms: exercise for physical health, mindfulness for present-moment awareness, or group activities for social connection. PESoMind integrates four components (physical activity, emotional expression, social connection, mindfulness) recognizing that adolescent wellbeing is multidimensional (6). These components function as mutually reinforcing rather than independent interventions. Physical movement generates energy, supporting emotional expression, facilitating social connection, and enabling mindfulness. Multiple entry points allow adolescents to engage through whichever component resonates most, then access others.

Prevention and Health Promotion: Traditional mental health interventions address symptoms after emergence. PESoMind targets prevention and promotion, building resilience before crisis. This aligns with public health frameworks emphasizing upstream interventions reaching broader populations (4). Low barriers (no cost, minimal time, no specialized training) position the framework for universal prevention rather than targeted treatment. This complements rather than replaces clinical care, serving populations who might not access clinical services.

## Theoretical contribution

PESoMind's theoretical contribution lies in integrating multiple evidence-based modalities through a single accessible activity. Rather than requiring adolescents to choose between separate interventions (exercise programs, mindfulness apps, support groups, or expressive therapies), the framework delivers all four simultaneously through dance. This bundling approach addresses a practical challenge in intervention selection: matching adolescents to appropriate modalities typically requires screening processes that themselves create access barriers. The multiple entry points design allows engagement through any component (physical movement, emotional expression, social connection, or mindfulness practice), with the others delivered concurrently. An adolescent drawn to physical activity receives emotional expression and social connection benefits alongside their primary interest. This may enable synergies not achievable through single modalities alone, though this hypothesis requires empirical testing.

## Conclusion

Adolescent mental health challenges continue to rise while access to support remains unequal. Addressing this requires more than expanding clinical capacity. It requires fundamentally rethinking what accessible mental health promotion looks like.

PESoMind illustrates one approach to accessibility-first design: integrating evidence-based components (physical activity, emotional expression, social connection, mindfulness) through an accessible modality (dance) while eliminating implementation barriers (time, cost, training, space). Preliminary evidence shows initial acceptability among adult stakeholders and reach to 500+ students via a digital platform. However, rigorous trials with adolescent participants, control conditions, and longitudinal assessment are essential to establish effectiveness.

As adolescent mental health challenges intensify, expanding the intervention options with more accessible and engaging approaches becomes even more urgent. PESoMind offers a model for equity-focused innovation in adolescent mental health promotion.

## Data Availability

The datasets presented in this study can be found in online repositories. The names of the repository/repositories and accession number(s) can be found in the article/supplementary material.
