# Correction: Reimagining accessibility in adolescent mental health: PESoMind, a dance-based framework for wellness promotion

**DOI:** 10.3389/fpubh.2026.1910333

**Published:** 2026-07-06

**Authors:** Tara Srinivasan, Afshan Khan

**Affiliations:** 1 Westlake High School, Austin, TX, United States; 2 Austin Family Psychiatry, Austin, TX, United States; 3 Department of Psychiatry & Behavioral Sciences, Dell Medical School, The University of Texas at Austin, Austin, TX, United States

**Keywords:** accessibility, adolescent mental health, dance intervention, digital health, health equity

In the published article, there was a mistake in the *M* value listed for social connectedness in the section **Proof-of-concept: SXSW EDU 2025 workshop**, *Outcomes*, paragraph number 1. The first sentence was displayed as:

“Participants reported consistently high ratings across all measured dimensions: energy (*M* = 9.17, *SD* = 0.83), mindfulness (*M* = 8.75, *SD* = 0.87), social connectedness (*M* = 8.67, *SD* = 1.07), and emotional state (*M* = 8.58, *SD* = 0.90).”

The correct sentence is:

“Participants reported consistently high ratings across all measured dimensions: energy (*M* = 9.17, *SD* = 0.83), mindfulness (*M* = 8.75, *SD* = 0.87), social connectedness (*M* = 7.25, *SD* = 1.07), and emotional state (*M* = 8.58, *SD* = 0.90)”.

The original version of this article has been updated.

